# Supplementary material for: Splice-Junction-Based Mapping of Alternative Isoforms in the Human Proteome
Source: Cell Rep. Author manuscript; Available in PMC 2020 Jan 15. (PMC6961840; doi:10.1016/j.celrep.2019.11.026)
Supplement: 2 [file NIHMS1546469-supplement-2.zip › DF1/PXD010154/Heart/Heart_69_MAPKAPK5_QVIEEQTTSHESTGGAK.pdf]

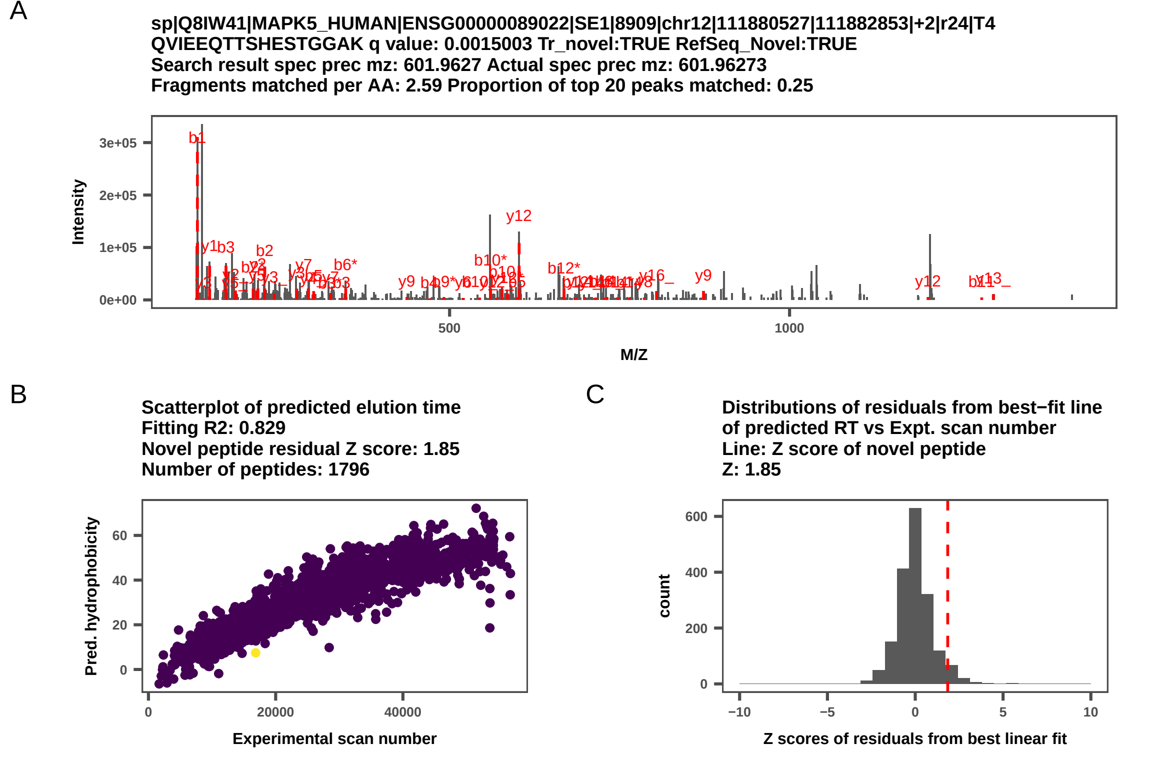
A
sp|Q8IW41|MAPK5_HUMAN|ENSG00000089022|SE1|8909|chr12|111880527|111882853|+2|r24|T4
QVIEEQTTSHESTGGAK q value: 0.0015003 Tr_novel:TRUE RefSeq_Novel:TRUE
Search result spec prec mz: 601.9627 Actual spec prec mz: 601.96273
Fragments matched per AA: 2.59 Proportion of top 20 peaks matched: 0.25
Intensity
3e+05
2e+05
1e+05
0e+00
b1
y12
b10*
b12*
b6*
y9
y12
M/Z
500
1000
B
Scatterplot of predicted elution time
Fitting R2: 0.829
Novel peptide residual Z score: 1.85
Number of peptides: 1796
Pred. hydrophobicity
60
40
20
0
Experimental scan number
0
20000
40000
C
Distributions of residuals from best−fit line
of predicted RT vs Expt. scan number
Line: Z score of novel peptide
Z: 1.85
count
600
400
200
0
Z scores of residuals from best linear fit
−10
−5
0
5
10
